# Screening of Proximal and Interacting Proteins in Rice Protoplasts by Proximity-Dependent Biotinylation

**DOI:** 10.3389/fpls.2017.00749

**Published:** 2017-05-12

**Authors:** Qiupeng Lin, Zejiao Zhou, Wanbin Luo, Maichun Fang, Meiru Li, Hongqing Li

**Affiliations:** ^1^Guangdong Provincial Key Lab of Biotechnology for Plant Development, South China Normal UniversityGuangzhou, China; ^2^Key Laboratory of Plant Resources Conservation and Sustainable Utilization, South China Botanical Garden, Chinese Academy of SciencesGuangzhou, China; ^3^Guangdong Provincial Key Laboratory of Applied Botany, South China Botanical Garden, Chinese Academy of SciencesGuangzhou, China

**Keywords:** BioID, protein–protein interactions, rice, protoplast, OsFD2

## Abstract

Proximity-dependent biotin identification (BioID), which detects physiologically relevant proteins based on the proximity-dependent biotinylation process, has been successfully used in different organisms. In this report, we established the BioID system in rice protoplasts. Biotin ligase *BirAG* was obtained by removing a cryptic intron site in the *BirA^∗^* gene when expressed in rice protoplasts. We found that protein biotinylation in rice protoplasts increased with increased expression levels of BirAG. The biotinylation effects can also be achieved by exogenous supplementation of high concentrations of biotin and long incubation time with protoplasts. By using this system, multiple proteins were identified that associated with and/or were proximate to OsFD2 *in vivo*. Our results suggest that BioID is a useful and generally applicable method to screen for both interacting and neighboring proteins in their native cellular environment in plant cell.

## Introduction

Proteins perform a vast array of functions within living organisms, including catalyzing enzymatical reactions, DNA replication, stimuli responding, and molecule transporting from one location to another. Most proteins carry out their functions together with other proteins in a protein complex. Protein–protein interactions are a hallmark of all essential cellular processes (Ellis, 2001; Tabaka et al., 2014). Thus, detection of protein–protein interaction *in vivo* is an essential part to understand the function of individual proteins at molecular level. Until now, many methods have been developed and used to screen and identify the interacting proteins, such as the yeast-two hybrid system (Y2H), the co-immunoprecipitation (Co-IP) and the bimolecular fluorescence complementation (BiFC), etc. Some methods can be merely used *in vitro*, which may not reflect the actual interaction inside cells, or suffer the loss of candidate proteins because of protein insolubility or transient or weak protein interactions (Roux et al., 2012).

Proximity-dependent biotin identification (BioID) method, which overcomes the above restrictions, has become a unique method to screen for physiologically relevant protein interactions in living cells (Roux et al., 2013; Rees et al., 2015). The Biotin ligase mutation BirA^∗^(R118G) is the essential and functional part of BioID. Wild-type BirA normally only transfers a biotin to a substrate bearing a specific recognition sequence. BirA^∗^ is promiscuous *in vivo*, it activates biotin for transferring in the absence of a substrate peptide within less than 10 nm around the BirA^∗^-tagged protein (Firat-Karalar and Stearns, 2015). After that, the biotinylated protein can be identified by western-blot or mass spectrometry (MS). BioID has been successfully used to screen the interacting or vicinal proteins in mammalian cells, such as probing the architecture of nuclear pore complex (Kim et al., 2014) and centrosome components (Firat-Karalar et al., 2014), identifying the substrates of ubiquitin E3 ligase (Coyaud et al., 2015), analyzing the interactions of virus-related proteins (Ritchie et al., 2015), and screening the interacting proteins in bacteria and tumors (Dingar et al., 2015; Batsios et al., 2016). However, the successful use of BioID in plants has not been reported.

In this report, *BirAG*, an improved version of *BirA^∗^* was obtained by removing a cryptic intron splitting site in *BirA*^∗^, and was expressed in rice cells. By studying factors that influence *BirAG* function in rice protoplasts, a simple and efficient BioID system was established in rice. We also investigated the efficiency of the method on the identification of proteins that associate with and/or are proximate to OsFD2, a protein that has been found to be involved in vegetative growth in rice (Tsuji et al., 2013).

## Materials and Methods

### Plasmids

*Escherichia coli BirA* was PCR amplified and mutated to R118G (BirA^∗^) by overlap extension PCR (Barker and Campbell, 1981). Products of both the *BirA* and *BirA^∗^* contain a 5′ *Bgl* II site and a 3′ *Bam* HI site. To obtain *BirAG*, a cryptic intron splicing site in *BirA^∗^* was changed by PCR-mediated mutation The *BirA*, *BirA*^∗^, and *BirAG* were digested with Bgl II and Bam HI, and inserted into pCAMBIA 1390 or pUbi1390 binary vectors digested with Bam HI. pUbi1390 is a vector derived from pCAMBIA1390, in which the CaMV35S promoter is replaced by a maize ubiquitin promoter. Target proteins EGFP, OsFD1, OsFD2, Hd3a and RCN1 were fused in frame with BirA, BirA^∗^ or BirAG using the restriction sites Bam HI and (or) Eco RI, with the target proteins in the C terminals. A FLAG tag was added in frame at the C terminals of different target proteins. All the PCR primers for the above manipulation and the coding sequence of *BirA*, *BirA*^∗^, or *BirAG* are listed in the Supplementary Document.

### Protoplasts Isolation and Transfection

Rice protoplasts isolation and transfection were performed as described (Zhang et al., 2011). Dehulled seeds of rice (*Oryza sativa* L.) cultivar “Zhonghua11” were sterilized and germinated on 1/2 MS medium for 8–12 days. The stem and sheath tissues of rice seedlings were used for protoplasts isolation. For protoplasts transfection, 10 μl of plasmids at a concentration of 1 μg/μl was used for 100 μl protoplasts (about 2 × 10^6^). After transformation, the protoplasts were collected by centrifugation and resuspended in 250 μl WI. For biotinylation experiments, WI contains exogenous biotin was added to the protoplasts.

### Western Blotting

About 1 × 10^6^ protoplasts were lysed in Laemmli SDS-sample buffer. Proteins were separated by SDS-PAGE and transferred to PVDF (Roche) membrane (Komatsu, 2015). Immunoblotting was performed (Liu et al., 2007) with mouse anti-FLAG (1:50,000; Abcam). For detection of biotinylated proteins, membranes were blocked in 3% casein in TBS with 0.05% Tween-20 and incubated in the same buffer with HRP-conjugated streptavidin (1:40,000; Invitrogen).

### Affinity Capture of Biotinylated Proteins

3 × 10^6^ protoplasts were incubated for 24 h in WI solution with 50 μM biotin. After three washes with 0.6 M mannitol solution, protoplasts were lysed according to Roux et al. (2012). The supernatant containing the extracted proteins were incubated with 600 μl Dynabeads (MyOne Streptavidin C1; Invitrogen) overnight at 4°C. Stringency washing of beads was performed according to Roux et al. (2012). Ten percentage of the beads were reserved for Western blot analysis. Bound proteins were removed from the magnetic beads with 50 μl of Laemmli SDS-sample buffer saturated with biotin and boiled at 98°C for 10 min.

### Protein Identification Using Mass Spectrometry

After stringency washing, the beads were washed six times with 50 mM ammonium bicarbonate (pH 8.3), and treated with 0.5 μg/μl TPCK-trypsin (Promega) 16 h at 37°C. The supernatant containing the tryptic peptides was collected and lyophilized.

The nano LC MS/MS analysis was carried out using an Orbitrap Fusion Tribrid (Thermo-Fisher Scientific, San Jose, CA, USA) mass spectrometer. The Orbitrap was interfaced with an UltiMate 3000 RSLC nano system (Thermo-Dionex, Sunnyvale, CA, USA). Each reconstituted fraction (2 μl) at a rate of 6 μl/min was injected onto a PepMap C18 trapping column (5 μm, 200 μm × 1 cm, Dionex). Then each fraction was separated on the PepMap C18 RP nano column (3 μm, 75 μm × 15 cm, Dionex), by a 135 min gradient from 4 to 36% ACN in 0.1% FA at 300 nl/min. The fractions were subjected to a 5-min ramp to 90% ACN-0.1% FA and a 10-min hold at 90% ACN-0.1% FA. The column was circulated with 4% ACN-0.1% FA for 11 min.

The separated peptides were detected by the Orbitrap fusion, which was operated in positive ion mode with the source temperature at 275°C and nanospray voltage set at 1.9 kV. The instrument used Ultramark 1621 for the FT mass analyzer as external calibration and the background polysiloxane ion signal at *m*/*z* 445.120025 as internal calibration. MS spectra were acquired across the mass range of 350–1550 m/z, with detection at a resolution of 120000 in the orbitrap mass analyzer, using 50 ms accumulation time per spectrum. Tandem mass spectra were recorded in high sensitivity mode (resolution >30000) and made by high energy collision dissociation (HCD) with the mass range of *m*/*z* 100–1000. In each cycle of data-dependent acquisition (DDA) analysis, following each survey scan, the 10 most intense multiply charged ions above a threshold ion count of 5000 were selected for fragmentation at a normalized collision energy of 40% and dynamic exclusion for 30 s. All data were acquired with Xcalibur 2.1 software (Thermo-Fisher Scientific).

### Protein Identification and Quantitation

Raw data files acquired from Fusion were converted into MGF files using Proteome Discoverer 1.4 (PD 1.4, Thermo). Subsequent database searches were carried out by Mascot Daemon (version 2.4.1, Matrix Science, Boston, MA, USA) for both protein identifications and TMT quantitation against the protein databases. The default Mascot search settings were as follows: one missed cleavage site by trypsin allowed with fixed IAA modification of cysteine, fixed 6-plex TMT modifications on Lys and N-terminal amines and variable modifications of methionine oxidation, deamidation of Asn and Gln residues, biotinylation of Lys residues. The peptide and fragment mass tolerance values were 10 ppm and 0.6 Da, respectively. To estimate the false discovery rate (FDR) for a measure of identification certainty in each replicate set, we employed the target-decoy strategy of Elias and Gygi (2007). Specifically, an automatic decoy database search was performed in Mascot by choosing the decoy checkbox in which a random sequence of database is generated and tested for raw spectra as well as the real database. To reduce the probability of false peptide identification, Percolator was performed by choosing the Percolator checkbox. The manufacturer’s recommended isotope correction factors were applied. Only peptides above “identity” were counted as identified, it was required that each confident protein identification involved at least two unique peptide identifications indicated in Mascot. The proteins identified within the same family were grouped.

### BiFC Analysis of Putative Vicinal Proteins in Rice Protoplasts

The putative protein genes to be identified were amplified with PCR and restriction-cloned (BamHI and/or EcoRI) into the p35S::cYFP vector, which in fusion with the C-terminal fragment of YFP at 3′ end. In the meanwhile, OsFD1 and OsFD2 gene were generated by PCR and restriction-cloned in the p35S::nYFP vector, which in fusion with the N-terminal fragment of YFP at 5′ end. All constructs were sequence-verified before transformation. The vectors were co-transformed into rice protoplasts, which were cultivated over night before live imaging. Pictures were acquired using LSM 710 confocal microscope (Zeiss, Jena, Germany). For studying the subcellular localization of individual protein, expression vectors containing GFP fused with different proteins were transformed into rice protoplasts, and the fluorescence were observed using the same confocal microscope.

### Yeast Two-Hybrid Analysis

Yeast two-hybrid analysis was performed using an AH109-GAL4 system (Clontech). PCR-amplified fragments of OsFD1 and OsFD2 was cloned into pGBKT7 (Y2H) to form the bait construct, and the fragments containing the candidate genes were inserted into pGADT7 to form the prey constructs. The bait and prey constructs, together with thermally denatured salmon sperm carrier DNA, were transformed into yeast strain AH109 according to the manufacturer’s instructions (Clontech). After that, the transformants were grown on various media at 28°C.

## Results

### Modification of BirA^∗^ Gene for Expressing in Rice Protoplasts

**FIGURE 1:**
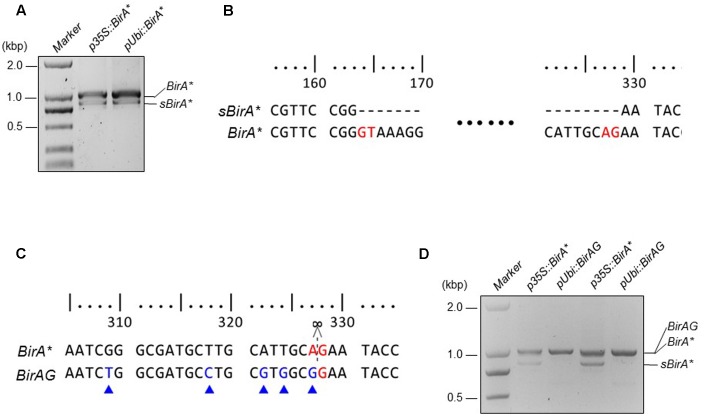
**Modified BirA^∗^(BirAG) and its transcripts in rice protoplasts. (A)** RT-PCR products of BirA^∗^ transcripts in rice protoplasts. sBirA^∗^ indicates the alternative spliced form of BirA^∗^. **(B)** Sequence alignment of sBirA^∗^ and BirA^∗^. Bases in red color indicate the intron splicing sites. **(C)** Sequence alignment of BirA^∗^ and BirAG. Bases in red color indicate splicing site and bases in blue color indicate codon optimized sites. **(D)** RT-PCR products of BirA^∗^ and BirAG transcripts in rice protoplasts. Rice protoplasts were transfected with the indicated plasmids and incubated overnight, total RNA was extracted from each sample and was used for RT-PCR. p35S, CaMV35S promoter; pUbi, Maize ubiquitin promoter.

To obtain the *BirA^∗^* gene, wild-type *BirA* gene from *E. coli* was amplified by PCR. To endow the biotin ligase with the function of biotinylating vicinal proteins, we created R118G BirA (BirA^∗^) by Overlap Extension PCR (Ho et al., 1989). Additional transcript (*sBirA^∗^*) was produced when *BirA^∗^* was expressed in rice protoplasts, indicating that alternative splicing of *BirA^∗^* occurred (**Figure 1A**). Sequencing of the two transcripts confirmed the alternative splicing, and the nucleotides from 164 to 328 from the coding region of *BirA^∗^* were removed in *sBirA^∗^*. The GU-AG splicing is a typical intron splicing mode in plant cell (**Figure 1B**). To avoid alternative splicing of *BirA^∗^* in rice cell, we created *BirAG* by using PCR-mediated site specific mutagenesis. We also optimized some codons inside the spliced region (**Figure 1C** and Supplementary Data Sheet 1). We next expressed *BirAG* in rice protoplasts, and observed a single transcript indicating that the cryptic sites for intron splicing in *BirA^∗^* has been removed (**Figure 1D**). The expression levels of both *BirA^∗^* and *BirAG* were higher when driven by the maize ubiquitin promoter than by the CaMV35S promoter (**Figures 1A,D**).

### Transient Expression of BirAG Can Biotinylate Proteins in Rice Protoplasts

Biotin identification method has been applied in different organism for identification of protein complex or interacting proteins successfully. In plants, protoplasts can be manipulated like mammalian cells and can be transformed efficiently. Thus, we tried to find whether BirAG can biotinylate proteins in transiently transformed rice protoplasts.

**FIGURE 2:**
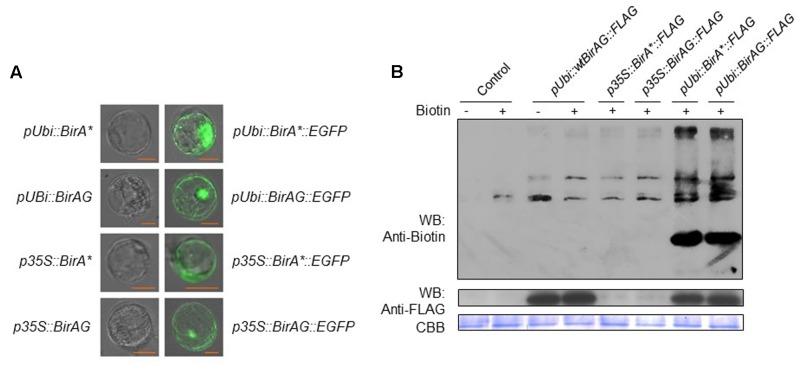
**BirAG can biotinylate itself and proteins in rice protoplasts. (A)** Expression and localization of BirA^∗^ and BirAG proteins in rice protoplasts. Plasmids used for protoplasts transfection were indicated. **(B)** Western-blot of biotinylated proteins in rice protoplasts after transient expression of BirA-FLAG, BirA^∗^-FLAG, and BirAG-FLAG, with or without biotin at 50 μM in protoplasts incubation solution. Different plasmids used for protoplasts transfection were indicated. Streptavidin-HRP and Anti-FLAG antibody were used for detection of biotinylated proteins and different BirA versions, respectively. Coomassie Brilliant Blue (CBB G-250) staining was used for monitoring the sample loading amount. p35S, CaMV35S promoter; pUbi, maize ubiquitin promoter; arrow indicate the biotinylated BirAG proteins.

First, we transformed rice protoplasts with four expression vectors of *p35S::BirA^∗^-EGFP*, *pUbi::BirA^∗^-EGFP, p35S::BirAG-EGFP*, and *pUbi::BirAG-EGFP*. After overnight incubation, we observed EGFP protein accumulation inside rice protoplasts, which indicates that BirAG protein can be produced efficiently in rice protoplasts. As maize ubiquitin promoter is much stronger than CaMV35S promoter in rice, we found brighter EGFP fluorescence in protoplasts transformed with *pUbi::BirA^∗^-EGFP* and *pUbi::BirAG-EGFP* (**Figure 2A**). As the majority of *BirA^∗^* transcripts produced in protoplasts are intact (**Figures 1A,D**), normal EGFP signals were also observed in protoplasts expressing the BirA^∗^-EGFP fusion proteins (**Figure 2A**). Together with the assay of RNA transcripts of these vectors in rice protoplasts, we conclude that higher expression of *BirAG* in RNA and protein level can be obtained in rice protoplasts by using maize ubiquitin promoter.

Next, we examined whether BirA^∗^ and BirAG could function in rice protoplasts to biotinylate proteins. Rice protoplasts were transformed with *pUbi::BirA-FLAG, p35S::BirA^∗^-FLAG*, *p35S::BirAG-FLAG, pUbi::BirA^∗^-FLAG*, *pUbi::BirAG-FLAG* and were incubated in WI containing 50 μM biotin for 24 h. Proteins were extracted from the protoplasts for western-blot assays of both biotinylated protein and BirA derived proteins. More biotinylated proteins were found in extracts from protoplasts transformed with *pUbi::BirA^∗^-FLAG* and *pUbi::BirAG-FLAG* than the control protoplasts transformed with *pUbi-BirA-FLAG* (**Figure 2B**). The *BirA^∗^* and *BirAG* protein levels were much lower in protoplasts transformed with constructs driven by the CaMV35S promoter than those by the maize ubiquitin promoter as indicated in the western blot with a FLAG tag antibody. The Coomassie Brilliant Blue (CBB G-250) staining showed that similar amount of proteins were loaded in each lane (**Figure 2B**).

### Factors Influencing BirAG Biotinylates Proteins in Rice Protoplasts

To examine whether *BirAG* expression level will influence the protein biotinylation, we performed the biotinylation assay in rice protoplasts transformed with *p35S::BirAG-FLAG* and *pUbi::BirAG-FLAG*, which expressed lower and higher levels of BirAG, respectively. Protoplasts transformed with the *pUbi::BirAG-FLAG* showed stronger biotinylated protein band than those transformed with the *p35S::BirAG-FLAG*. This result indicates that higher BirAG protein level are more effective in protein biotinylation. The self-biotinylation of the transformed BirAG was also detected (**Figure 2B**).

**FIGURE 3:**
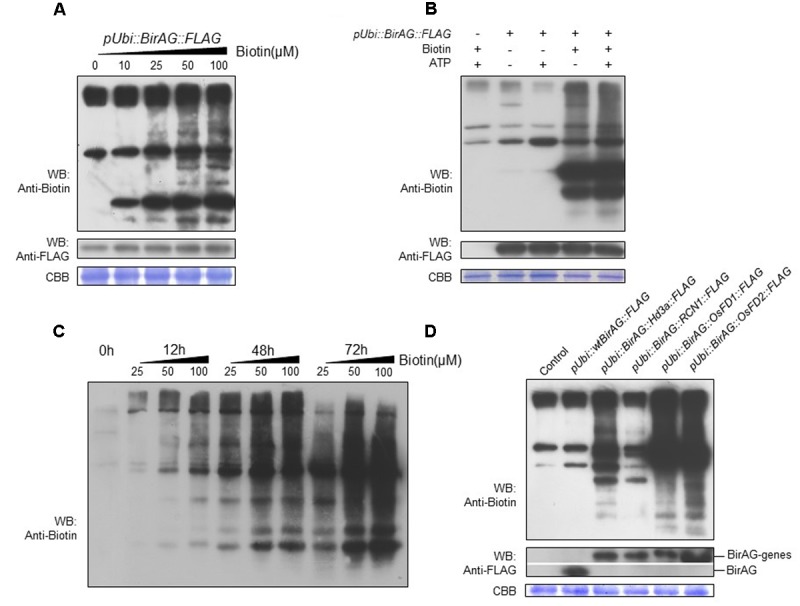
**Factors influencing BirAG on biotinylating proteins in rice protoplasts. (A)** Biotinylation of proteins increases with the biotin concentration in protoplasts incubation buffer. **(B)** Influence of ATP on protein biotinylation in rice protoplasts, ATP was added into the protoplasts incubation buffer at the concentration of 10 μM. **(C)** Influence of protoplasts incubation time and biotin concentrations on protein biotinylation in rice protoplasts. **(D)** BirAG fused with different target proteins can biotinylated proteins in rice protoplasts. Different treatment and plasmids used for protoplasts transfection were indicated. Streptavidin-HRP and Anti-FLAG antibody were used for detection of biotinylated proteins and BirAG, respectively. Coomassie Brilliant Blue (CBB G-250) staining was used for monitoring the sample loading amount. pUbi, maize ubiquitin promoter; arrows indicate the biotinylated BirAG proteins.

For biotinylation reaction *in vitro*, both biotin and ATP are needed (Choi-Rhee et al., 2004). Thus, we investigate the effects of different concentrations of exogenous biotin and (or) ATP supplement on biotinylation reaction in rice protoplasts that express BirAG protein. The results showed that exogenous biotin addition was necessary for the reaction and the concentration of 50 μM was sufficient for achieving optimal biotinylation (**Figure 3A**). The supplement of 10 μM ATP in the presence of 50 μM biotin had no significant effects (**Figure 3B**). We also compared the effects of different incubation time on protein biotinylation and found that protein biotinylation increased with longer incubation time. More biotinylated protein bands appeared on the blot after 48 h (**Figure 3C**), but further increasing the incubation time to 72 h results no additional increase of the biotinylated proteins, suggesting the decrease of protoplasts viability or the saturation of protein biotinylation.

To test whether this method is suitable for identifying protein complex or neighboring proteins *in vivo*, we constructed expression vectors by fusing BirAG with different target proteins including OsFD1 (*Os09g0540800*), OsFD2 (*Os06g0720900*), Hd3a (*Os06g0157700*), and OsRCN1 (*Os11g0152500*), proteins involved in rice flowering and vegetative growth (Taoka et al., 2013; Sun et al., 2014). Western blotting showed that all these fusion proteins can function in rice protoplasts and produced biotinylated protein bands which are different from the non-transformed and the wild-type BirA control (**Figure 3D**). These results indicate that more proteins are biotinylated besides the endogenous naturally existed biotinylated proteins in rice cell. Interestingly, the banding patterns of the biotinylated proteins among the four constructs were different, suggesting that different target proteins may have their own vicinal partners (**Figure 3D**). Taken together, *BirAG* driven by maize ubiquitin promoter, with exogenous biotin at 50 mM, and a protoplasts incubation time of 24–48 h were optimal for achieving promiscus protein biotinylation in rice. These results indicate that target proteins fused with BirAG might be useful for probing neighboring proteins or protein complex in rice protoplasts.

### Identifying Vicinal Proteins of OsFD2 in Rice Protoplasts

**FIGURE 4:**
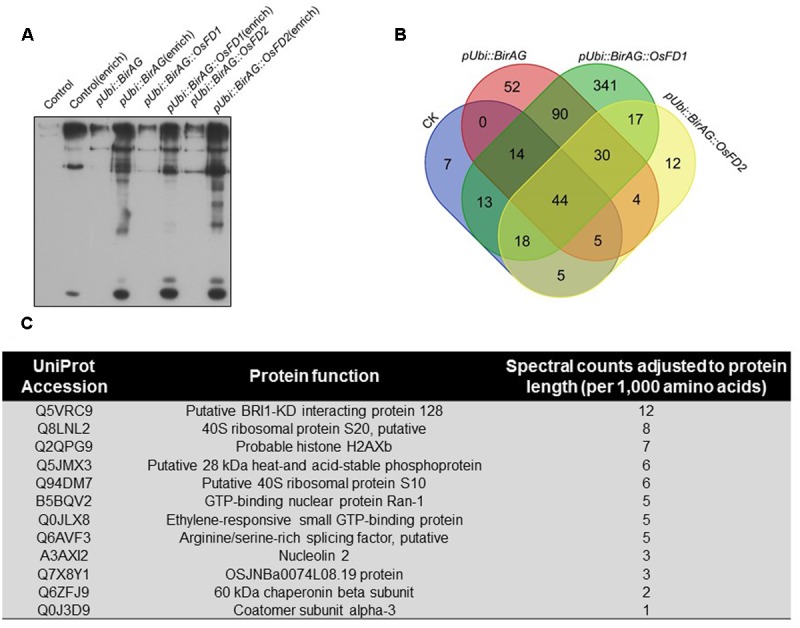
**Proximity-dependent promiscuous protein biotinylation by pUbi::BirAG-OsFD2. (A)** Enrichment of biotinylated proteins from rice protoplasts transformed with BirAG fused with different target proteins. Proteins were extracted from transfected protoplasts and were subjected to streptavidin-coupled beads for protein enrichment. Samples of 10 μl before or after enrichment were used for loading. **(B)** Schematic Venn diagram of the proteins identified by MS in protoplasts transfected with pUbi::BirA, pUbi::BirAG, pUbi::BirAG-OsFD1, pUbi::BirAG-OsFD2. The detailed protein lists were shown in the Supplementary Tables. **(C)** Putative proteins which are proximal to OsFD2 and their predicted function. MS identified proteins from pUbi::BirAG-OsFD2 transfected protoplasts which are different from that in pUbi::BirA, pUbi::BirAG, pUbi::BirAG-OsFD1 are listed.

To investigate the feasibility of BioID for screening vicinal proteins in rice, we selected OsFD2 as an example. OsFD2 is a transcription factor involving in plant vegetative growth. Tsuji et al. (2013) showed that this protein interacted with 14-3-3 for cytoplast retention. We transformed rice protoplasts with *pUbi::BirAG-OsFD2*. After incubation of the protoplasts for 24 h, proteins were extracted. The protoplasts transformed with *pUbi::WTBirA*, *pUbi::BirAG*, and *pUbi::BirAG-FD1* were used as controls. The biotinylated proteins were enriched by streptavidin-coupled magnetic beads, 1/10 of the beads were boiled in the SDS-PAGE sample buffer and the released proteins were examined by western blot analysis. The rest of the beads were used for on-bead protein digestion with trypsin and the peptides were profiled by MS analysis. The enrichment of biotinylated proteins by streptavidin-coupled beads were shown in **Figure 4A**.

The MS results showed that 135 proteins were identified in *Ubi::BirAG-OsFD2* transformed protoplasts (Supplementary Table S4). And 108, 239 and 567 proteins were identified in the *pUbi::WTBirA*, *pUbi::BirAG*, and *pUbi::BirAG-FD1*, respectively (Supplementary Tables S1–S3). The most abundant background proteins identified in *pUbi::BirA* and *pUbi::BirAG* are the naturally biotinylated carboxylases and associated enzymes such as Acetyl-CoA carboxylase 2, Acetyl-CoA carboxylase 1, and Methylcrotonoyl-CoA carboxylase subunit alpha, which largely coincided with the intensely staining bands. We also found background binding of plastid proteins, nuclear proteins involved in mRNA processing, as well as of ribosomal proteins (Supplementary Tables). To eliminate non-specifically biotinylated proteins, background proteins in *pUbi::BirA* and *pUbi::BirAG* were subtracted from the *pUbi::BirAG-OsFD2* protein list. As OsFD2 is the homolog of OsFD1 and they function differently in plant development, the list of potential interactors with OsFD2 was further narrowed down by eliminating the proteins identified in *pUbi::BirAG-OsFD1* (**Figure 4B**). Finally, twelve putative OsFD2 neighboring proteins were identified. These proteins belong to different categories including putative BRI1-KD interacting protein 128, GTP binding proteins, histone and ribosomal proteins etc. (**Figure 4C**).

OsFD2 is a transcription factor, which had been reported to form a complex with 14-3-3 and function in rice vegetative growth (Tsuji et al., 2013). When studying the datasets, we found one G-box binding protein (Q0J5J5, 14-3-3 like) in all the four protein lists. We also found that additional two 14-3-3 like proteins (Q2R2W2 and Q10E23) in BirAG-OsFD1 dataset (Supplementary Table S4). As both OsFD1 and OsFD2 has been found to form complex with 14-3-3 proteins, the above results suggest that additional 14-3-3s as OsFD1 interactors can be found by BioID. However, it is uncertain whether OsFD2 does interact with the 14-3-3 like (Q0J5J5), as the latter was found in different control datasets (Supplementary Tables). To investigate whether proteins in BirAG-OsFD2 list are really proximal to OsFD2, eight proteins (after subtracting from only WTBirA dataset) were selected for further analysis. Among them, three proteins Q5VMJ3, Q5JMX3, and Q0JLX8 (Q5VMJ3 also in BirAG-OsFD1 list) gave positive BiFC results.

**FIGURE 5:**
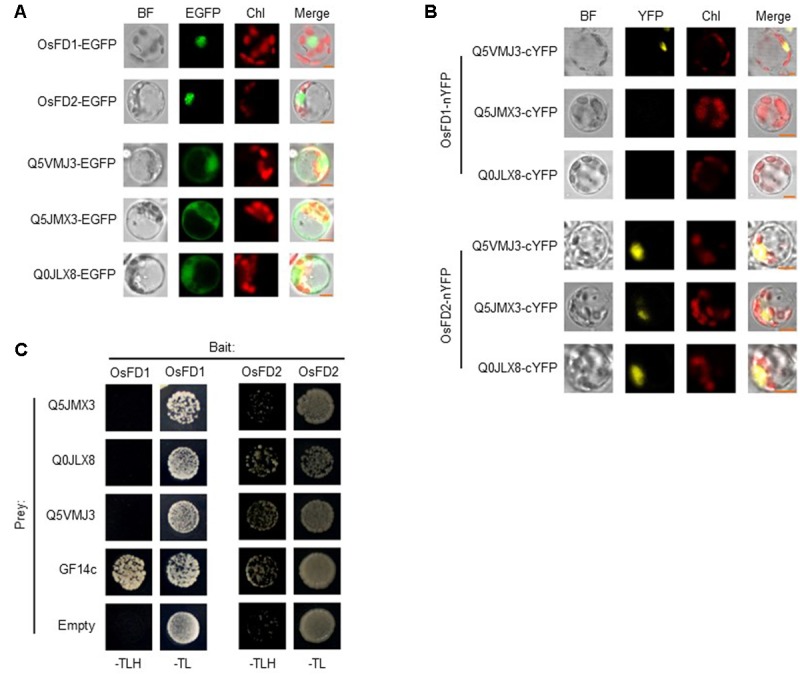
**Confirmation of proteins proximal to OsFD2 or OsFD1 by BiFC and Y2H. (A)** Localization of OsFD1, OsFD2, Q5VMJ3, Q5JMX3, and Q0JLX8 in rice protoplasts, respectively. Proteins were fused with EGFP and were transformed into rice protoplasts. BF, bright field; GFP, green fluorescence signal; Chl, chloroplasts auto-fluorescence; Merge, merged image. **(B)** Representative BiFC results of three proteins proximal to OsFD1 or OsFD2 in rice protoplasts. OsFD1 or OsFD2 was fused with nYFP and three proteins were fused with cYFP. OsFD1-nYFP or OsFD2-nYFP was co-transformed into rice protoplasts with either of the three cYFP fused proteins, and subjected to confocal analysis. BF, bright field; YFP, yellow fluorescence signal; Chl, chloroplasts auto-fluorescence; Merge, merged image. **(C)** Examination of OsFD1 or OsFD2 interacting with three proteins by yeast two hybrid. OsFD1 or OsFD2 was used as bait. Empty and GF14c were used as negative and positive controls, respectively.

For BiFC assays, the subcellular localization of OsFD1 and OsFD2 in rice protoplasts was investigated, and the two proteins are localized mainly in nucleus (**Figure 5A**). The localization of OsFD2 is somewhat different from a former study, which used protoplasts derived from rice OC cells (Tsuji et al., 2013). The underlying reason could be that the localization of OsFD2 in mesophyll cell is different from that in OC cell. The three putative OsFD2 neighboring proteins Q5VMJ3, Q5JMX3, and Q0JLX8 were all located in both cytoplasm and nucleus (**Figure 5A**). BiFC results showed that OsFD2 is proximal to all these three proteins in nucleus, while OsFD1 is proximal only to Q5VMJ3, which correlated well with the BioID results (**Figure 5B**). Q5VMJ3 (*Os06g0152100*) is an actin monomer binding protein, it binds to actin and affects the structure of the cytoskeleton. The actin dynamics has been found to be involved in protein transporting into nucleus (Hirano and Matsuura, 2011). Q5JMX3 (*Os01g0752800*) is a putative heat and acid stable phosphoprotein, the homolog of this protein in human is PADP1, which involves in cell proliferation and signaling (Fischer and Schubert, 1996). Q0JLX8 (*Os01g0558600*), a Ras-related RIC1, belongs to the small GTPase superfamily and Rab family, which functions in vesicle budding, motility and fusion (Uchimiya et al., 1993). As the biotin ligase has a labeling radius of about 10 nm, proteins isolated by BioID may interact with the target protein directly or indirectly. The other five proteins showed negative results in BiFC assays suggest that these proteins might indirectly interact with OsFD2 or they are non-specifically biotinylated proteins. Further studies with Y2H showed that OsFD2 might interact with Q0JLX8 and Q5VMJ3, while there is no interaction was observe between OsFD1 and either of the three proteins (**Figure 5C**). As Y2H detects direct and stable protein interaction in a non-physiological condition, the proteins in physical proximity inside a living cell may not give a positive result by this method.

## Discussion

Biotin identification is a novel approach for screening proximal and potentially interacting proteins. It has been successfully applied to study a variety of proteins and processes in mammalian cells and unicellular eukaryotes and has been shown to be particularly suited to the study of insoluble or inaccessible cellular structures and for detecting weak or transient protein associations (Varnaitė and MacNeill, 2016).

In this report, we established the BioID system in rice protoplasts. We modified the BirA^∗^ gene by removing a cryptic intron site in its coding region (*BirAG*), and thus ensured the effective expression of full length protein. The parameters influencing the biotin ligase functioning in rice cell such as gene expression level, biotin concentration and protoplast incubation time were studied, which forms the basis of using BioID system in plant cell. As rice protoplasts can be easily obtained from germinated seedlings and can be transformed with high efficiency, the BioID method based on rice protoplasts has potential to study protein interactome in plants.

Similar to mammalian cells, biotinylating activity of BirAG in plant cells can be controlled in different aspects (Roux et al., 2012). First, *BirAG* expression level is important to achieve optimal protein biotinylation, and the expression can be controlled by using different promoters. Second, the concentration of exogenous biotin supplement can be used to control biotinylation reaction in plant cells. Although plant cell can synthesize biotin, the free biotin in plant cell might not be enough for promiscuous protein biotinylation. Finally, the reaction can be controlled by incubating at different time to control protein biotinylation effects. The above characteristics provide multiple choices to control the biotinylation effects for further improvement of the system in plants.

Biotin identification has been successfully applied to characterize stable structural assemblies previously, such as the nuclear envelope and pore complex (Roux et al., 2012; Kim et al., 2014) or centrosomes (Firat-Karalar and Stearns, 2015). Recently, the method was used to study proteins involved in NMD, a more transient and dynamic complexes (Schweingruber et al., 2016). In this report, we applied this method to study proteins interacting or proximal to transcription factor OsFD2 in rice, which is synthesized in cytoplasm and transported to the nucleus as a DNA binding protein. OsFD2 has been found interacting with 14-3-3, and together with Hd3a, form a complex in regulating plant vegetative growth (Tsuji et al., 2013). As a 14-3-3 like protein (Q0J5J5) was found in different control experiments, it could not be identified as an OsFD2 proximal protein in our experiments. However, we detected two additional 14-3-3 like proteins (Q2R2W2 and Q10E23) in OsFD1 dataset, which suggests that proteins proximal to OsFD1 can be identified by BioID. Hd3a forms a complex with OsFD2, which are bridged by 14-3-3. We did not find Hd3a in the OsFD2 protein dataset. The reason might be that there is no direct contact between Hd3a and OsFD2 or the very low expression level of Hd3a in seedlings (Hd3a as a florigen mainly expresses in mature tissue while the protoplasts were isolated from young seedlings). After subtracting of the proteins in different controls from OsFD2 list, we obtained twelve putative proteins (**Figure 4C**). Among them, Q0JLX8 and Q5VMJ3 were confirmed by BiFC and/or Y2H to be proximal to OsFD2.

Mass spectrometry data from BioID experiments generally comprise of a large list of potential interactome components, non-specifically biotinylated proteins, as well as abundant background proteins. In the control experiment with *pUbi::WTBirA*, naturally existed biotinylated proteins in rice cell, such as acetyl CoA carboxylase and methylcrotonoyl CoA carboxylase (Supplementary Table S1), and a large number of abundant proteins such as the ribosomal, histone, and Rubisco proteins were trapped down by the streptavidin coupled magnetic beads. As promiscuous biotinylation could not be catalyzed by the wild type BirA, the background are probably come from non-specifically binding proteins to streptavidin (proteins can still be detected by MS after stringent washing). Similar results have also been found in mammalian cells (Roux et al., 2012). The large amount of background proteins often bring difficulties in validation of the truly proteins that are proximal to target protein. Generally, three measures can be adopted to solve this problem: (1) Select proteins that were isolated at highest abundance from MS data. The highest abundance is a semiquantitative approach and may reflect the association between two proteins; (2) combine BioID with quantitative proteomics techniques such as SILAC, iTRAQ, or SWATH to eliminate non-specifically biotinylated proteins; (3) perform appropriate controls for subtracting background proteins (Varnaitė and MacNeill, 2016).

In our work, three different controls were used for subtracting background proteins. When studying the subtracting effects of each control, we found that the number of putative OsFD2 proximal proteins left were 62,30,12, respectively, after removing the proteins from the control lists (Supplementary Table S5). Eight proteins from the OsFD2 protein list after subtraction of WTBirA proteins were selected for BiFC analysis, and three of them gave positive results. This indicated that single control may not be enough to remove background proteins. Further subtraction with the BirAG control reduced the OsFD2 protein list to 30, while the three proteins were still on the list. Finally, we found that Q5VMJ3 appeared on both OsFD1 and OsFD2 proteins lists and this was proved by the BiFC assay. The other two proteins exclusively belong to the OsFD2 list were confirmed by BiFC and/or Y2H (Supplementary Table S5).

Although our studies proved that BioID could be used for screening potential protein interactome in rice protoplasts, further improvement of this method is needed in the following aspects: (1) achieving more specific biotinylation of neighboring proteins by controlling the temporal and spatial expression of BirAG fused target protein; (2) eliminating non-specifically biotinylated proteins or background proteins by combining BioID with quantitative proteomics techniques, and design multiple controls; (3) studying protein interactomes in different cell, tissue and organ types by using stable transgenic plants. This system, either to be used independently or together with other existing methods, will benefit the studies of protein networks in plant.

## Conclusion

In this report, we obtained a modified biotin ligase gene *BirAG*, which can be expressed in rice protoplasts with high efficiency. We studied factors influencing on the BirAG biotinylation function in rice protoplasts and established the BioID system in rice. By using this system, multiple proteins that associate with OsFD2 but not OsFD1, the closest homolog of OsFD2, were identified *in vivo*. This system, either to be used independently or together with other existing methods, will benefit the studies of protein networks in rice.

## Author Contributions

HL designed and carried out the project. ML directed the study. QL carried out the studies on factors influencing BirAG function in rice protoplasts and protein identification. ZZ performed the BiFC analysis. WL and MF performed gene cloning and rice protoplasts transformation. ML and HL wrote the manuscript. All authors read and approved the final manuscript.

## Conflict of Interest Statement

The authors declare that the research was conducted in the absence of any commercial or financial relationships that could be construed as a potential conflict of interest.
