# Supplementary material for: Screening of Proximal and Interacting Proteins in Rice Protoplasts by Proximity-Dependent Biotinylation
Source: Front Plant Sci. 2017 May 12;8:749. doi: 10.3389/fpls.2017.00749 (PMC5427108; doi:10.3389/fpls.2017.00749)
Supplement: Supplementary file 6 [file Data_Sheet_1.DOCX]

**Supplementary Doc**. PCR primers and sequences of BirA, BirA* and BirAG

1. *Primers for amplification and modification of BirA (restriction sites are underlined, red colored font indicates mutated base for BirA*)*

BirAFw: AATTAGATCTATCATGAAGGATAACACCGT

BirARe: AATTGGATCCGGATTTTTCTGCACTACGCAGGGA

BirAMF:TCCGACCACGACCACCACGACCAGCATGCT

BirAMR:AGCATGCTGGTCGTGGTGGTCGTGGTCGGA

BirASphI:AATTGCATGCTGGTATTCCGCCACGCAGGCATCGCCAGATTTAAGCTC

1. *Primers for amplification of OsFD2 and other protein genes for BiFC*

OsFD2Fw:AATTGGATCCATGGCGAACTACCACCACCAGGAGT

OsFD2Re:AATTGAATTCTCAGAATTGAGTTGATGAAGTTCGTCGC

OsFD2ERe(for EGFP vector)：AATTGAATTCAAAGAATTGAGTTGATGAAGTTCGTCGC

Q5VMJ3Fw：AATTGGATCCATGTCGTGGCAGGCGTACGTCG

Q5VMJ3Re：AATTGAATTCTTAGCAACCCTGCTCGATCAGATAATCG

Q5VMJ3ERe(for EGFP vector)：AATTGAATTCAAAGCAACCCTGCTCGATCAGATAATCG

Q5JMX3Fw：AATTGGATCCATGGGCAGGGGGAAGTTCAAGG

Q5JMX3Re：AATTGAATTCTTATTTGCGTGCTTCAGCCTTCCTCTG

Q5JMX3Re(for EGFP vector)：AATTGAATTCAAATTTGCGTGCTTCAGCCTTCCTCTG

Q0JLX8Fw：AATTGGATCCATGAATCCCGAGTATGACTACCTCTTCAAGC

Q0JLX8Re：AATTGAATTCTCAAGAGCAGCAGCTGCTTTGCTG

Q0JLX8Re(for EGFP vector)：AATTGAATTCAAAAGAGCAGCAGCTGCTTTGCTG

1. *Sequence of BirA, BirA* and BirAG*

**Sequence of wild type BirA coding region (restriction site SphI is underlined)**

ATGAAGGATAACACCGTGCCACTGAAATTGATTGCCTGTTAGCGAACGGTGAATTTCACTCTGGCGAGCAGTTGGGTGAAACGCTGGGAATGAGCCGGGCGGCTATTAATAAACACATTCAGACACTGCGTGACTGGGGCGTTGATGTCTTTACCGTTCCGGGTAAAGGATACAGCCTGCCTGAGCCTATCCAGTTACTTAATGCTAAACAGATATTGGGTCAGCTGGATGGCGGTAGTGTAGCCGTGCTGCCAGTGATTGACTCCACGAATCAGTACCTTCTTGATCGTATCGGAGAGCTTAAATCGGGCGATGCTTGCATTGCAGAATACCAGCATGCTGGTCGTGGTCGTCGTGGTCGGAAATGGTTTTCGCCTTTTGGCGCAAACTTATATTTGTCGATGTTCTGGCGTCTGGAACAAGGCCCGGCGGCGGCGATTGGTTTAAGTCTGGTTATCGGTATCGTGATGGCGGAAGTATTACGCAAGCTGGGTGCAGATAAAGTTCGTGTTAAATGGCCTAATGACCTCTATCTGCAGGATCGCAAGCTGGCAGGCATTCTGGTGGAGCTGACTGGCAAAACTGGCGATGCGGCGCAAATAGTCATTGGAGCCGGGATCAACATGGCAATGCGCCGTGTTGAAGAGAGTGTCGTTAATCAGGGGTGGATCACGCTGCAGGAAGCGGGGATCAATCTCGATCGTAATACGTTGGCGGCCATGCTAATACGTGAATTACGTGCTGCGTTGGAACTCTTCGAACAAGAAGGATTGGCACCTTATCTGTCGCGCTGGGAAAAGCTGGATAATTTTATTAATCGCCCAGTGAAACTTATCATTGGTGATAAAGAAATATTTGGCATTTCACGCGGAATAGACAAACAGGGGGCTTTATTACTTGAGCAGGATGGAATAATAAAACCCTGGATGGGCGGTGAAATATCCCTGCGTAGTGCAGAAAAATCC

**Sequence of BirA*coding region (blue colored bases indicate the cryptic intron splicing sites, shaded bases indicate intron sequences, red colored base indicates mutation R118G, restriction site SphI is underlined)**

ATGAAGGATAACACCGTGCCACTGAAATTGATTGCCTGTTAGCGAACGGTGAATTTCACTCTGGCGAGCAGTTGGGTGAAACGCTGGGAATGAGCCGGGCGGCTATTAATAAACACATTCAGACACTGCGTGACTGGGGCGTTGATGTCTTTACCGTTCCGGGTAAAGGATACAGCCTGCCTGAGCCTATCCAGTTACTTAATGCTAAACAGATATTGGGTCAGCTGGATGGCGGTAGTGTAGCCGTGCTGCCAGTGATTGACTCCACGAATCAGTACCTTCTTGATCGTATCGGAGAGCTTAAATCGGGCGATGCTTGCATTGCAGAATACCAGCATGCTGGTCGTGGTGGTCGTGGTCGGAAATGGTTTTCGCCTTTTGGCGCAAACTTATATTTGTCGATGTTCTGGCGTCTGGAACAAGGCCCGGCGGCGGCGATTGGTTTAAGTCTGGTTATCGGTATCGTGATGGCGGAAGTATTACGCAAGCTGGGTGCAGATAAAGTTCGTGTTAAATGGCCTAATGACCTCTATCTGCAGGATCGCAAGCTGGCAGGCATTCTGGTGGAGCTGACTGGCAAAACTGGCGATGCGGCGCAAATAGTCATTGGAGCCGGGATCAACATGGCAATGCGCCGTGTTGAAGAGAGTGTCGTTAATCAGGGGTGGATCACGCTGCAGGAAGCGGGGATCAATCTCGATCGTAATACGTTGGCGGCCATGCTAATACGTGAATTACGTGCTGCGTTGGAACTCTTCGAACAAGAAGGATTGGCACCTTATCTGTCGCGCTGGGAAAAGCTGGATAATTTTATTAATCGCCCAGTGAAACTTATCATTGGTGATAAAGAAATATTTGGCATTTCACGCGGAATAGACAAACAGGGGGCTTTATTACTTGAGCAGGATGGAATAATAAAACCCTGGATGGGCGGTGAAATATCCCTGCGTAGTGCAGAAAAATCC

**Sequence of BirAG coding region (green colored bases indicate codon differences between BirA* and BirAG, blue colored bases indicate changed intron splicing site, restriction site SphI is underlined)**

ATGAAGGATAACACCGTGCCACTGAAATTGATCGCCCTGTTAGCGAACGGTGAATTTCACTCTGGCGAGCAGTTGGGTGAAACGCTGGGAATGAGCCGGGCGGCTATTAATAAACACATTCAGACACTGCGTGACTGGGGCGTTGATGTCTTTACCGTTCCGGGTAAAGGATACAGCCTGCCTGAGCCTATCCAGTTACTTAATGCTAAACAGATATTGGGTCAGCTGGATGGCGGTAGTGTAGCCGTGCTGCCAGTGATTGACTCCACGAATCAGTACCTTCTTGATCGTATCGGAGAGCTTAAATCTGGCGATGCCTGCGTGGCGGAATACCAGCATGCTGGTCGTGGTGGTCGTGGTCGGAAATGGTTTTCGCCTTTTGGCGCAAACTTATATTTGTCGATGTTCTGGCGTCTGGAACAAGGCCCGGCGGCGGCGATTGGTTTAAGTCTGGTTATCGGTATCGTGATGGCGGAAGTATTACGCAAGCTGGGTGCAGATAAAGTTCGTGTTAAATGGCCTAATGACCTCTATCTGCAGGATCGCAAGCTGGCAGGCATTCTGGTGGAGCTGACTGGCAAAACTGGCGATGCGGCGCAAATAGTCATTGGAGCCGGGATCAACATGGCAATGCGCCGTGTTGAAGAGAGTGTCGTTAATCAGGGGTGGATCACGCTGCAGGAAGCGGGGATCAATCTCGATCGTAATACGTTGGCGGCCATGCTAATACGTGAATTACGTGCTGCGTTGGAACTCTTCGAACAAGAAGGATTGGCACCTTATCTGTCGCGCTGGGAAAAGCTGGATAATTTTATTAATCGCCCAGTGAAACTTATCATTGGTGATAAAGAAATATTTGGCATTTCACGCGGAATAGACAAACAGGGGGCTTTATTACTTGAGCAGGATGGAATAATAAAACCCTGGATGGGCGGTGAAATATCCCTGCGTAGTGCAGAAAAATCC
